# Supplementary material for: Influence of study shift on the interrelationships among chronobiological factors, health practices, and anthropometry in adolescents
Source: PLoS One. 2025 May 30;20(5):e0322617. doi: 10.1371/journal.pone.0322617 (PMC12124575; doi:10.1371/journal.pone.0322617)
Supplement: S1 Table — (DOCX) [file pone.0322617.s001.docx]

S1 Table. Model fit indices

|  | **Models** | | | | | |
| --- | --- | --- | --- | --- | --- | --- |
| **SEM** | **1** | **2** | **3** | **4** | **5** | **6** |
| χ2 (df) | 94.104 (68) | 93.040 (72) | 125.336 (83) | 105.514 (87) | 71.273 (38) | 58.096 (41) |
| p-value χ2 | 0.020 | 0.048 | <0.002 | 0.086 | <0.001 | 0.040 |
| χ2/(df) | 1.384 | 1.292 | 1.510 | 1.213 | 1.875 | 1.417 |
| CFI | 0.963 | 0.940 | 0.939 | 0.945 | 0.943 | 0.933 |
| TLI | 0.955 | 0.930 | 0.927 | 0.937 | 0.915 | 0.906 |
| SRMR | 0.036 | 0.038 | 0.033 | 0.039 | 0.034 | 0.036 |
| RMSEA (CI90%) | 0.027  (0.011-0.040) | 0.027  (0.002-0.042) | 0.031  (0.019-0.042) | 0.023  (0.000-0.037) | 0.041  (0.026-0.055) | 0.032  (0.007-0.050) |

SEM: Structural equation modeling. χ2: Chi-square (p-value > 0.05); χ2/df: Ratio between Chi-square and degrees of freedom (< 5); CFI: Comparative fit index (≥0.90); TLI: Tucker–Lewis index (> 0.90); RMSEA: Root mean square error fit index (≤ 0.06); SRMR: Standardized root mean square residual (<0.08)
