# Supplementary material for: Influence of study shift on the interrelationships among chronobiological factors, health practices, and anthropometry in adolescents
Source: PLoS One. 2025 May 30;20(5):e0322617. doi: 10.1371/journal.pone.0322617 (PMC12124575; doi:10.1371/journal.pone.0322617)
Supplement: S2 Table — (DOCX) [file pone.0322617.s002.docx]

S2 Table. Indirect and total effects in models 1, 3 and 5, stratified by morning study shift

|  | **Model 1** | | | | **Model 3** | | | | **Model 5** | | | |
| --- | --- | --- | --- | --- | --- | --- | --- | --- | --- | --- | --- | --- |
|  | **Indirect** | | **Total** | | **Indirect** | | **Total** | | **Indirect** | | **Total** | |
| **Indirect effects **** | **β** | **p** | **β** | **p** | **β** | **p** | **β** | **p** | **β** | **p** | **β** | **p** |
| Chronotype → SJL→ Complete meals | 0.046 | 0.587 | -0.002 | 0.976 | - | - | - | - | - | - | - | - |
| Chronotype → SJL→ Unhealthy foods | - | - | - | - | -0.06 | 0.505 | 0.130 | 0.095 | - | - | - | - |
| Chronotype → SJL→ Vegetables | - | - | - | - | - | - | - | - | -0.051 | 0.420 | -0.022 | 0.631 |
| Chronotype → SJL→ Fruits | - | - | - | - | - | - | - | - | -0.044 | 0.481 | 0.037 | 0.405 |
| SDW → SJL→ Complete meals | <-0.001 | 0.805 | -0.012 | 0.87 | - | - | - | - | - | - | - | - |
| SDW → SJL→ Unhealthy foods | - | - | - | - | <-0.001 | 0.717 | -0.01 | 0.91 | - | - | - | - |
| SDW → SJL→ Vegetables | - | - | - | - | - | - | - | - | <-0.001 | 0.785 | 0.035 | 0.424 |
| SDW → SJL→ Fruits | - | - | - | - | - | - | - | - | <-0.001 | 0.772 | 0.044 | 0.317 |
| Chronotype → SDW→ Complete meals | 0.002 | 0.877 | -0.045 | 0.685 | - | - | - | - | - | - | - | - |
| Chronotype → SDW→ Unhealthy foods | - | - | - | - | 0.002 | 0.902 | 0.192 | 0.082 | - | - | - | - |
| Chronotype → SDW→ Vegetables | - | - | - | - | - | - | - | - | -0.006 | 0.432 | 0.022 | 0.783 |
| Chronotype → SDW→ Fruits | - | - | - | - | - | - | - | - | -0.008 | 0.332 | 0.073 | 0.344 |
| PA → SDW→ Complete meals | <-0.001 | 0.944 | 0.059 | 0.307 | - | - | - | - | - | - | - | - |
| PA → SDW→ Unhealthy foods | - | - | - | - | <-0.001 | 0.941 | -0.040 | 0.524 | - | - | - | - |
| PA → SDW→ Vegetables | - | - | - | - | - | - | - | - | <-0.001 | 0.929 | 0.114 | 0.009* |
| PA → SDW→ Fruits | - | - | - | - | - | - | - | - | <-0.001 | 0.929 | 0.147 | <0.001* |
| TST → SDW→ Complete meals | <0.001 | 0.869 | -0.170 | 0.009* | - | - | - | - | - | - | - | - |
| TST → SDW→ Unhealthy foods | - | - | - | - | <0.001 | 0.909 | 0.121 | 0.096 | - | - | - | - |
| TST → SDW→ Vegetables | - | - | - | - | - | - | - | - | -0.001 | 0.629 | -0.076 | 0.083 |
| TST → SDW→ Fruits | - | - | - | - | - | - | - | - | -0.001 | 0.585 | -0.072 | 0.108 |
| Chronotype → SDW→ SJL | 0.002 | 0.781 | 0.833 | <0.001* | 0.002 | 0.677 | 0.833 | <0.001* | 0.002 | 0.762 | 0.833 | <0.001* |
| PA → SDW→ BMI/age | <0.001 | 0.933 | 0.044 | 0.332 | <0.001 | 0.932 | 0.044 | 0.334 | <0.001 | 0.928 | 0.022 | 0.635 |
| TST→ PA → SDW | <0.001 | 0.934 | -0.031 | 0.515 | <0.001 | 0.932 | -0.032 | 0.503 | <0.001 | 0.929 | -0.031 | 0.513 |
| Chronotype → PA → Complete meals | <-0.001 | 0.964 | -0.048 | 0.674 | - | - | - | - | - | - | - | - |
| Chronotype → PA → Unhealthy foods | - | - | - | - | <0.001 | 0.960 | 0.190 | 0.092 | - | - | - | - |
| Chronotype → PA → Vegetables | - | - | - | - | - | - | - | - | <-0.001 | 0.970 | 0.028 | 0.726 |
| Chronotype → PA → Fruits | - | - | - | - | - | - | - | - | <-0.001 | 0.970 | 0.081 | 0.300 |
| PA → Complete meals → BMI/age | 0.002 | 0.678 | 0.045 | 0.318 | - | - | - | - | - | - | - | - |
| PA → Unhealthy foods → BMI/age | - | - | - | - | 0.001 | 0.656 | 0.045 | 0.321 | - | - | - | - |
| PA → Vegetables → BMI/age | - | - | - | - | - | - | - | - | 0.007 | 0.216 | 0.028 | 0.529 |
| PA → Fruits → BMI/age | - | - | - | - | - | - | - | - | 0.019 | 0.037* | 0.040 | 0.381 |
| SDW → Complete meals → BMI/age | <-0.001 | 0.877 | -0.111 | 0.013* | - | - | - | - | - | - | - | - |
| SDW → Unhealthy foods → BMI/age | - | - | - | - | <-0.001 | 0.909 | -0.113 | 0.012* | - | - | - | - |
| SDW → Vegetables → BMI/age | - | - | - | - | - | - | - | - | 0.002 | 0.489 | -0.117 | 0.010* |
| SDW → Fruits → BMI/age | - | - | - | - | - | - | - | - | 0.005 | 0.351 | -0.114 | 0.012* |

*Significant p-value ** The arrow (→) indicates a direct relationship between variables. The model reflects the mediated effect of variable 1 on variable 3, passing through variable 2. The table presents the indirect effects (variable 1 → variable 2 → variable 3) and the total effects (direct effect + indirect effect). All direct effects are shown in Table 3.

Fit indices used - χ2: Chi-square (p-value > 0.05); χ2/df: Ratio between chi-square and degrees of freedom (< 5); CFI: Comparative fit index (≥0.90); TLI: Tucker–Lewis index (> 0.90); RMSEA: Root mean square error fit index (≤ 0.06); SRMR: Standardized root mean square residual (<0.08). Legends: SDW: Sleep duration on school days; BMI/age: Body mass index by age; SJL: Social jet lag; TST: Total screen time; PA: Physical activity level
