# Supplementary material for: Influence of study shift on the interrelationships among chronobiological factors, health practices, and anthropometry in adolescents
Source: PLoS One. 2025 May 30;20(5):e0322617. doi: 10.1371/journal.pone.0322617 (PMC12124575; doi:10.1371/journal.pone.0322617)
Supplement: S3 Table — (DOCX) [file pone.0322617.s003.docx]

S3 Table. Indirect and total effects in models 2, 4 and 6, stratified by afternoon study shift

|  | **Model 2** | | | | **Model 4** | | | | **Model 6** | | | |
| --- | --- | --- | --- | --- | --- | --- | --- | --- | --- | --- | --- | --- |
|  | **Indirect** | | **Total** | | **Indirect** | |  | | **Indirect** | | **Total** | |
| **Indirect effects **** | **β** | **p** | **β** | **p** | **β** | **p** | **β** | **p** | **β** | **p** | **β** | **p** |
| Chronotype → SJL→ Complete meals | 0.036 | 0.354 | -0.342 | <0.001* | - | - | - | - | - | - | - | - |
| Chronotype → SJL→ Unhealthy foods | - | - | - | - | 0.018 | 0.642 | -0.009 | 0.911 | - | - | - | - |
| Chronotype → SJL→ Vegetables | - | - | - | - | - | - | - | - | 0.080 | 0.005* | -0.106 | 0.068 |
| Chronotype → SJL→ Fruits | - | - | - | - | - | - | - | - | 0.044 | 0.070 | -0.179 | <0.001* |
| SDW → SJL→ Complete meals | -0.020 | 0.363 | 0.17 | 0.001* | - | - | - | - | - | - | - | - |
| SDW → SJL→ Unhealthy foods | - | - | - | - | -0.009 | 0.640 | -0.054 | 0.441 | - | - | - | - |
| SDW → SJL→ Vegetables | - | - | - | - | - | - | - | - | -0.044 | 0.016* | 0.026 | 0.599 |
| SDW → SJL→ Fruits | - | - | - | - | - | - | - | - | -0.024 | 0.076 | 0.090 | 0.050* |
| Chronotype → SDW→ Complete meals | 0.033 | 0.045* | -0.345 | <0.001* | - | - | - | - | - | - | - | - |
| Chronotype → SDW→ Unhealthy foods | - | - | - | - | -0.006 | 0.556 | -0.032 | 0.723 | - | - | - | - |
| Chronotype → SDW→ Vegetables | - | - | - | - | - | - | - | - | 0.009 | 0.241 | -0.177 | 0.005* |
| Chronotype → SDW→ Fruits | - | - | - | - | - | - | - | - | 0.015 | 0.075 | -0.207 | <0.001* |
| PA → SDW→ Complete meals | 0.005 | 0.671 | -0.068 | 0.325 | - | - | - | - | - | - | - | - |
| PA → SDW→ Unhealthy foods | - | - | - | - | <-0.001 | 0.724 | -0.004 | 0.955 | - | - | - | - |
| PA → SDW→ Vegetables | - | - | - | - | - | - | - | - | 0.001 | 0.683 | 0.094 | 0.061 |
| PA → SDW→ Fruits | - | - | - | - | - | - | - | - | 0.002 | 0.664 | 0.165 | 0.001* |
| TST → SDW→ Complete meals | -0.025 | 0.098 | -0.004 | 0.963 | - | - | - | - | - | - | - | - |
| TST → SDW→ Unhealthy foods | - | - | - | - | 0.004 | 0.577 | 0.361 | 0.002* | - | - | - | - |
| TST → SDW→ Vegetables | - | - | - | - | - | - | - | - | -0.007 | 0.298 | -0.060 | 0.237 |
| TST → SDW→ Fruits | - | - | - | - | - | - | - | - | -0.011 | 0.097 | 0.021 | 0.674 |
| Chronotype → SDW→ SJL | -0.032 | 0.035* | 0.418 | <0.001* | -0.031 | 0.036* | 0.431 | <0.001* | -0.034 | 0.028* | 0.429 | <0.001* |
| PA → SDW→ BMI/age | -0.001 | 0.700 | 0.028 | 0.563 | -0.002 | 0.684 | 0.032 | 0.512 | -0.002 | 0.676 | 0.033 | 0.513 |
| TST→ PA → SDW | <0.001 | 0.975 | -0.098 | 0.049* | <0.001 | 0.970 | -0.097 | 0.049* | <0.001 | 0.969 | -0.099 | 0.045* |
| Chronotype → PA → Complete meals | 0.006 | 0.388 | -0.372 | <0.001* | - | - | - | - | - | - | - | - |
| Chronotype → PA → Unhealthy foods | - | - | - | - | <0.001 | 0.966 | -0.026 | 0.774 | - | - | - | - |
| Chronotype → PA → Vegetables | - | - | - | - | - | - | - | - | -0.008 | 0.212 | -0.195 | 0.002* |
| Chronotype → PA → Fruits | - | - | - | - | - | - | - | - | -0.014 | 0.149 | -0.237 | <0.001* |
| PA → Complete meals → BMI/age | 0.007 | 0.406 | 0.036 | 0.452 | - | - | - | - | - | - | - | - |
| PA → Unhealthy foods → BMI/age | - | - | - | - | <-0.001 | 0.965 | 0.033 | 0.491 | - | - | - | - |
| PA → Vegetables → BMI/age | - | - | - | - | - | - | - | - | -0.010 | 0.146 | 0.025 | 0.621 |
| PA → Fruits → BMI/age | - | - | - | - | - | - | - | - | 0.010 | 0.257 | 0.045 | 0.354 |
| SDW → Complete meals → BMI/age | -0.025 | 0.224 | -0.083 | 0.122 | - | - | - | - | - | - | - | - |
| SDW → Unhealthy foods → BMI/age | - | - | - | - | 0.001 | 0.761 | -0.077 | 0.153 | - | - | - | - |
| SDW → Vegetables → BMI/age | - | - | - | - | - | - | - | - | -0.007 | 0.275 | -0.084 | 0.119 |
| SDW → Fruits → BMI/age | - | - | - | - | - | - | - | - | 0.007 | 0.294 | -0.069 | 0.203 |

*Significant p-value** The arrow (→) indicates a direct relationship between variables. The model reflects the mediated effect of variable 1 on variable 3, passing through variable 2. The table presents the indirect effects (variable 1 → variable 2 → variable 3) and the total effects (direct effect + indirect effect). All direct effects are shown in Table 3.

Fit indices used - χ2: Chi-square (p-value > 0.05); χ2/df: Ratio between chi-square and degrees of freedom (< 5); CFI: Comparative fit index (≥0.90); TLI: Tucker–Lewis index (> 0.90); RMSEA: Root mean square error fit index (≤ 0.06); SRMR: Standardized root mean square residual (<0.08). Legends: SDW: Sleep duration on school days; BMI/age: Body mass index by age; SJL: Social jet lag; TST: Total screen time; PA: Physical activity level
