# Supplementary material for: Influence of study shift on the interrelationships among chronobiological factors, health practices, and anthropometry in adolescents
Source: PLoS One. 2025 May 30;20(5):e0322617. doi: 10.1371/journal.pone.0322617 (PMC12124575; doi:10.1371/journal.pone.0322617)
Supplement: Attachment — Submitted filename: Response to Reviewers.docx [file pone.0322617.s005.docx]

Rio de Janeiro, March 22nd, 2025

Dear Editor,

We sincerely appreciate the insightful comments and constructive feedback provided by the reviewers on our manuscript titled "Influence of study shift on the interrelationships among chronobiological factors, health practices, and anthropometry in adolescents". We carefully considered all suggestions and have made substantial revisions to address the concerns raised. Below, we provide a detailed response to each comment, outlining the modifications incorporated into the **Revised Manuscript using Track Changes**. Additionally, we made necessary adjustments to align with PLOS ONE's style requirements. These include:

- Replacing "Figure" with "Fig.";
- Renaming all supporting information files, changing "Supplementary Tables 1, 2, and 3" to "S1, S2, and S3 Tables";
- Removing supporting tables from the main manuscript and submitting them as separate files.

We appreciate the time and consideration given to our work and look forward to your further feedback and that of the reviewers.

Best regards,

The authors.

**REVIEWER 1**

1. In Table 2 there is text that is written in Portuguese language.

**Response:** Thank you for your feedback. We have corrected the text in Table 2, ensuring that all content is now presented in English as required.

2. Please develop the limitation section including the reliance on self-reported data (e.g., food practices, screen time, and sleep duration) that may introduce bias or inaccuracies.

**Response:** Thank you for your feedback. We have revised the limitations section to provide a more detailed discussion of the potential biases and inaccuracies associated with self-reported data. These modifications can be found between lines 506–517.

**REVIEWER 2**

1. The cross-sectional design limits the ability to infer causality, which you acknowledge; however, further discussion on this limitation and its implications for interpreting the results would improve the manuscript.

**Response**: Thank you for your valuable feedback. In response, we have expanded the discussion on the limitations of our cross-sectional study design, addressing its implications for causal inference and interpretation of results. These revisions can be found between lines 491–492 and 499–505.

2. The reliance on self-reported measures for variables such as sleep duration, food practices, and screen time introduces potential bias. It might be useful to discuss how these biases could affect your findings or consider supplementing the questionnaire data with objective measures in future studies.

**Response**: Thank you for your feedback. We have revised the limitations section to further elaborate on the potential biases associated with self-reported data and their possible impact on our findings. Additionally, we have acknowledged the value of incorporating objective measurement tools in future research. These adjustments can be found between lines 506–517.

3. Although the formation of latent variables for food practices is innovative, the rationale behind the grouping of certain items - particularly the separate treatment of vegetables and fruits - could be explained more thoroughly to clarify why these items did not load with other healthy eating indicators.

**Response**: Thank you for your valuable observation. We recognize the importance of clarifying the separation of vegetables and fruits from other healthy eating indicators in the analysis. To address this, we have incorporated an additional explanation between lines 123 and 129.

In our model, latent variables require at least three indicators. Although fruits and vegetables were initially assigned to the same factor in the exploratory factor analysis, they formed a group with only two variables, making it methodologically inadequate to create a latent variable. Therefore, they were analyzed separately in the structural equation model.

Forcing fruits and vegetables to load into Factor 1, which comprises breakfast, lunch, dinner, and beans, did not yield meaningful results, as they did not present satisfactory factor loadings (≥ 0.3) in the confirmatory factor analysis for either school shift. This suggests that these variables had low correlation with Factor 1 and did not adequately represent its theoretical construct.

A possible explanation for this result is the study's setting: the federal educational institution provides free, high-quality meals daily, which may contribute to lesser differentiation of fruit and vegetable consumption. Furthermore, unlike beans and main meals, fruits and vegetables may be consumed in different contexts, such as snacks, and their inclusion in main meals depends on individual habits and availability. This partly explains their allocation to Factor 3 instead of Factor 1. As a result, Factor 1 was renamed from "healthy food practices" to "complete meals," while Factor 2 remained "unhealthy foods," and fruits and vegetables were analyzed as independent variables.

4. While the structural equation models appear robust, a more detailed presentation of the model fit indices and any modifications made based on the “modification indices” would enhance transparency.

**Response**: We appreciate your feedback. To improve transparency regarding the modeling process, we have clarified the description of the modification indices in the Statistical Analysis section of Materials and Methods (lines 229-232) and provided a more detailed description of the model fit indices in the corresponding results table (lines 283-290). These revisions ensure a clearer presentation of the methodological decisions taken and the robustness of the structural equation models.
